# The German RECAP questionnaire: linguistic validation and cognitive debriefing in German adults with self-reported atopic eczema and parents of affected children

**DOI:** 10.1186/s41687-021-00285-2

**Published:** 2021-01-21

**Authors:** Michaela Gabes, Christina Tischer, Anne Herrmann, Laura Howells, Christian Apfelbacher

**Affiliations:** 1 Institute of Social Medicine and Health Economics, Otto-von-Guericke-University Magdeburg, Magdeburg, Germany; 2 Medical Sociology, Department of Epidemiology and Preventive Medicine, University of Regensburg, Regensburg, Germany; 3 Centre of Evidence Based Dermatology, University of Nottingham, Nottingham, UK

**Keywords:** Atopic dermatitis, Atopic eczema, Long-term control, Cross-cultural translation, Content validity

## Abstract

**Background:**

Recap of atopic eczema (RECAP) is a patient-reported outcome measure (PROM) assessing eczema control. Long-term control of eczema is one of the four core outcome domains for atopic eczema trials. This instrument has been recently developed in the UK.

**Objective:**

This study aimed to translate the English RECAP into German and test its content validity in a German population with self-reported atopic eczema.

**Methods:**

A six-step procedure including two forward and one backward translations, two consensus decisions and an expert review was performed to obtain a German version of RECAP. We conducted semi-standardized cognitive interviews with adults with atopic eczema (*n* = 7) and parents having children affected by this disease (*n* = 5). A “think-aloud” method was used and aspects of comprehensibility, comprehensiveness and relevance according to the COnsensus-based Standards for the selection of health Measurement INstruments (COSMIN) criteria were examined. Interviews were coded using qualitative content analysis.

**Results:**

No particular linguistic problems were encountered during forward-backward translation. Minor wording changes were made as required. The title was adjusted to a more familiar German term of the disease (which is ‘*Neurodermitis*’). The recall period was rephrased from ‘over the last week’ to ‘over the last seven days’ since there was a different cultural understanding of the time frame. Regarding content validity, the items of the German RECAP were considered to be comprehensible, comprehensive and relevant for the participants and parents of affected children. The participants understood the instruction and considered the one-week recall period and the response options as appropriate.

**Conclusions:**

A German version of RECAP that is linguistically equivalent to the original version is now available but further assessment of its measurement properties is needed.

## Background

Atopic eczema (also called atopic dermatitis) is a pruritic skin disease often having a relapsing and remitting course and usually starting in early childhood. It affects up to 20% of children and up to 3% of adults [9]. The global HOME (Harmonising Outcome Measures for Eczema) initiative (www.homeforeczema.org) agreed on a core outcome set (COS), a minimum of outcomes that should be measured in every clinical eczema trial. ‘Long-term control of eczema’ is one of four domains of the COS [12]. No instrument measuring this construct in atopic eczema had been available. For this reason, a new outcome measurement instrument, named Recap of atopic eczema (RECAP), was developed and validated [5]. At the HOME VII meeting in Tokyo RECAP and another instrument, the Atopic Dermatitis Control Tool (ADCT) [10, 13], were both voted as core outcome instruments for the long-term domain. In order to further validate RECAP and increase its international applicability, linguistically validated translations with adequate content validity are needed. Content validity (i.e. the degree to which the content of a PROM is an adequate reflection of the construct to be measured) [8] is considered to be the most important measurement property. A PROM with its items, response options and instructions should be relevant, comprehensive, and comprehensible with respect to the construct of interest and the target population [7].

RECAP is a 7-item patient- or caregiver-reported instrument capturing the experience of eczema control. The patient and the caregiver version have been developed simultaneously to ensure that this instrument can be applied across all age groups. RECAP was developed in the UK, is free to access and use and can be completed quickly [5].

This study pursued two aims: 1) to linguistically validate RECAP in German language and 2) to test its content validity in a German population with self-reported atopic eczema.

## Methods

The whole translation process was conducted in accordance with the ISPOR Principles of Good Practice for the Translation and Cultural Adaptation Process for Patient-Reported Outcomes (PRO) Measures [16]. Before the translation process started, concepts were defined using a monolingual English dictionary (Oxford Advanced Learners Dictionary, 7th edition) to reduce personal interpretations of the translators and to facilitate an objective and precise translation. The translation process included six steps: two independent forward translations, a reconciliation, a backward translation by a third person, a harmonization meeting and an expert review. All three translators were German native speakers from Germany and fluent in English. Especially the backward-translator has been living in an English-speaking country for many years, however, this person was not a native English speaker. The backward translation was done blindly. It was reviewed by one of the forward-translators and harmonized within the whole research team. At the end, the German translation was reviewed by an expert, native-German and fluent in English, who has been working in the field of atopic eczema for many years. The entire translation process took approximately 3 months. The study was approved by the ethics committee of the University of Regensburg (file number: 19-1521-101).

In a second step, the translated version was cognitively debriefed in adults with self-reported atopic eczema and parents of affected children. Participants were recruited online via Facebook-groups for people affected by atopic eczema. A 10€-Amazon voucher was provided as an incentive. A topic guide (see Table 1) for the assessment of content validity was inspired by another content validity study [1] and developed in accordance with the COnsensus-based Standards for the selection of health Measurement INstruments (COSMIN) criteria for good content validity [14]. We conducted semi-standardized cognitive face-to-face or phone interviews with adults with atopic eczema (*n* = 7) and parents having children affected by this disease (*n* = 5). Probing techniques and a “think-aloud” method were used to capture the thoughts of the participants and potential difficulties when answering the questions [3, 15]. Questions on the comprehensibility of the title, the instruction, the items and the response options, on the comprehensiveness of the scale and the relevance of the single items were asked. Furthermore, we queried for the appropriateness of the recall period and for other suggestions for improvement. All interviews were audio-recorded and transcribed verbatim. Qualitative content analysis was used to code the single interviews [6]. Data was coded by MG and coding was further discussed and consented with LH and CA. Finally, the cognitive debriefing results were reviewed and discussed within the research team and the German RECAP version was finalized.

**Table 1:** Topics covered in the interview guide [1, 14]

Topics	Questions
General impression of the questionnaire	What were your feelings/thoughts when completing the questionnaire?
Comprehensibility	How comprehensible was the instruction to you? How comprehensible were the questions to you? How comprehensible were the response options to you? Are there any questions which should be phrased in a more specific manner?
Relevance	Are there any questions which are redundant, repeated or very similar?
Comprehensiveness	Do important aspects of eczema control lack in the questionnaire?
Response options	Are the response options appropriate?
Recall period	Is the recall period of “over the last week” appropriate?
Suggestions for improvement	Do you have any suggestions to improve the questionnaire?

## Results

The adult participants had a mean age of 35.14 years with a range from 17 to 48. Six of the seven participants were female (85.7%). Three participants got atopic eczema before their first year of life, one suffers from the disease since early childhood (at the age of ±2 years), one from middle childhood (at the age of ±6 years), one from puberty (at the age of ±12 years) and one from adulthood (at the age of ±21 years). Participating parents were all female and had children (male: 3, female: 2) from 3 months to 3 years. All children got atopic eczema within their first half year of life. Most of the mothers told that they were well informed about eczema. One mother did not know much about eczema since her child had only suffered from the disease for 4 weeks. No further interviews were conducted after theoretical saturation [11] was reached and no new aspects, neither for the adult nor for the child version, were generated. According to the COSMIN group, a sample size of 4–6 is considered to be adequate, a sample size of ≥7 is considered to be very good [14]. One interview took 13 min on average including the completion of the questionnaire and additional questions.

The general impressions of the questionnaire were positive. No participants suggested there were major problems and none expressed negative feelings about any of the questions.

### Comprehensibility

In general, most of the questions were easy to understand. Some problems emerged with item 3 (“Over the last week, on how many days has your skin been intensely itchy because of your eczema?”), item 5 (“Over the last week, how much has your eczema been getting in the way of day to day activities?”) and item 6 (“Over the last week, on how many days has your eczema affected how you have been feeling?”) and a more serious problem arose with item 7 (“Over the last week, how acceptable has your eczema been to you?”). Twenty-nine percent of the adults and 60% of the parents found the term ‘acceptable’ difficult to understand (see Table 2). They would have preferred something like ‘to cope with the disease’ or ‘to manage the disease’, suggesting that a verb instead of an adjective would work better. Furthermore, 43% of the adults and 80% of the parents didn’t understand or didn’t judge the title as suitable (for both versions “Recap for atopic eczema patients”). The German equivalent of ‘atopic eczema’ was perceived to be too medical and was less familiar for the participants. They would have preferred the more common name of the disease, ‘*Neurodermitis*’. Another aspect mentioned was the fact that the title of the child version was not appropriate since it was not evident that the questions refer to the child’s disease.

**Table 2:** Percentage of participants reporting comprehensibility of the title, instructions, items, and response options

	Adult version	Child version	Examples
Title	57%	20%	*“Reading ‘atopic eczema’ I would ask myself what that means […]*.*”, ID1, adult* *“This ‘atopic eczema’ I don’t know.”, ID8, adult* *“I know the term, this ‘atopic eczema’. But I think for the general public, this is too medical. I would rather say ‘skin disease’., ID3, parent*
Instruction	100%	100%	*“For me, this is really clear.”, ID4, adult*
Item 1	100%	100%	*“It is very comprehensible, this question.”, ID3, parent*
Item 2	100%	100%	*“This [question] was very well asked.”, ID8, adult*
Item 3	71%	60%	*“Intensely is somehow a thing of feeling. It was itchy that she cried, but not that she scratches herself raw. Well, it was strong, but it might have been even stronger.”, ID12, parent*
Item 4	100%	100%	*“I think this question is comprehensible to every person affected by eczema because sleep problems are very present when having these complaints.”, ID2, adult*
Item 5	86%	60%	*“Yes, this question was hard to answer, day-to-day activities with a baby, this is limited. […] I didn’t know what to tick because some things such as changing the child was really hard or bathing the child was a disaster because scratching started immediately. However, I could go for a walk with him, which is a day-to-day activity as well. […] It would be good to distinguish between day-to-day activities regarding care, rituals and sleep and leisure activities, social life* etc.*”, ID7, parent*
Item 6	71%	80%	*“Somehow phrased in a difficult way. […] I would make the question easy.”, ID3, parent*
Item 7	71%	40%	*“I do not fully understand what ‘acceptable’ means. I have to accept it either way because I have to live with the disease. I really don’t know what to answer. […] I think, with ‘acceptable’ you assume that you have a choice. […] But having atopic eczema is a fact that I have to accept.”, ID2, adult* *“Do I want to accept the disease? I don’t want to, I have to.”, ID5, adult*
Response options	100%	100%	*“I like that.”, ID8, adult*

### Relevance

All items were considered to be relevant by the participants *(“There was nothing redundant.“, ID4, adult*). Two participants (18%) spent time questioning if both item 2 (“Over the last week, on how many days has your skin been itchy because of your eczema?”) and 3 (“Over the last week, on how many days has your skin been intensely itchy because of your eczema?”) were required since they were really similar, but they concluded after re-reading that keeping both items was justified.

### Comprehensiveness

Two participants (20%) missed a question on restrictions due to the current treatment. Especially when having children, one mother felt less control over her child’s disease when she had to think about the extreme care for her child (having all the products such as cremes or special food all the time with her). Another two participants (20%) wished to include a question on whether they are well attended by their doctor. Having a confident contact person gave them the feeling of having more control over the disease. One mother (10%) missed a question on the parent sleep and an additional question on the intensity of itch and not just on the frequency. Two participants (20%) missed a question on comorbidities such as allergies or acute infections since especially for item 4 of the child version (“Over the last week, how much do you think your child’s sleep been disturbed because of their eczema?”), parents could not decide what was responsible for the child’s bad sleep (*“I couldn’t answer that (item 4) because she is having a bronchitis at the moment and I can’t decide: is it the itching or the coughing.”, ID12, parent*). And one participant (10%) would have wished a question on diet.

### Appropriateness of the response options

Eighty-three percent of the participants were happy with five response options. One participant would have liked to have two more options and another participant liked the amount of response options (except for item 1: too many), but would have preferred numerical values instead of words.

### Recall period

The recall period of 1 week was considered to be appropriate by 91% of the participants (one participant perceived it to be too long). However, 18% of the participants had problems with the formulation of the recall period since it was not completely clear to them whether they should consider the last 7 days or the last week from Monday to Sunday, especially when the interview was conducted towards the end of the current week.

*“When answering the questions towards the end of the week, this is maybe too long. Because this was then two weeks ago and it always depends on what day [you answer the questions].”, ID3, parent*

*“Considering last week, there I ask myself ‘the whole last week’? Because today it’s Thursday. From now or the last week?”, ID9, adult*

### Suggestions of improvement

One participant suggested to rather ask about the intensity of itch instead of frequency (item 2). Two participants had problems with the German translation of ‘eczema’ which goes more in the direction of the English ‘rash’. The would have preferred the German equivalent of ‘eczema’ or more general ‘disease’ since e.g. dryness of the skin is not included in ‘rash’ according to those participants. Another mother had a problem with the German neuter possessive pronoun which is identical to the masculine possessive pronoun.

There was no specific question regarding the layout of the questionnaire, but one participant expressed the wish to have additional boxes to tick instead of just words.

The following changes were applied:

- The title of RECAP was adjusted to ‘*Fragebogen für Patienten mit Neurodermitis*’ (adult version) and ‘*Fragebogen für Eltern mit Kindern mit Neurodermitis*‘ (child version). Item 7 was revised. The adjective ‘acceptable’ was expressed using the German translation of the verb ‘to cope with’ which was proposed by three interviewees. The corresponding response options of item 7 were slightly revised. Gender-specific possessive pronouns of the child version were removed. The recall period was revised from ‘over the last week’ to ‘over the last seven days’ since this was considered to be a cultural difference between English and German. In English, ‘over the last week’ and ‘over the last 7 days’ can be used equally, however in German, ‘over the last week’ is more likely to be interpreted from Monday to Sunday of the last week.

## Discussion

We assessed in a sample of 7 adults with atopic eczema and 5 parents with children with a atopic eczema the content validity of the German version of RECAP. This German version of RECAP that is content valid and linguistically equivalent to the original version is now available (upon request from the corresponding author). It is ready for the assessment of its measurement properties. In general, only minor cross-cultural problems were identified and resolved. All items of RECAP were considered to comprehensible, relevant and mostly comprehensive. Also, the instruction and response options were very well understood by the study participants.

Facebook proved to be a good and quick method to recruit participants since the main effort consisted in the posts and the appointment with the participants. Many people responded to the four posts and within 4 weeks, all interviews were conducted. Most of the participants were really open and enjoyed to help with their experiences. A strength of this study is the a priori developed topic guide which was in accordance with the COSMIN criteria for good content validity. To our knowledge, this is the first study to provide a German version of RECAP. A limitation of this online recruitment strategy is the bias towards online-active participants. We did not get any mid-aged (> 50 years) or old participants (> 65 years) since they are probably less active on the internet [4]. Furthermore, we only recruited in atopic eczema specific Facebook groups that automatically excluded those who did not belong to those groups. Another observable bias goes towards female participants. This is probably due to the fact that mostly mothers care for their children’s’ eczema [2].

This study included only native speakers and interviewees from Germany. We did not include any people from other German-speaking countries, such as Austria or Switzerland. For the use of the German RECAP in other German-speaking countries than Germany, further cognitive debriefing interviews are needed.

## Conclusion

With this study, we created a German version of RECAP that is linguistically equivalent to the original version and cognitively debriefed in Germany. The German RECAP is now available for use, but further assessment of its measurement properties is needed.

## Data Availability

The datasets used and analyzed during the current study are available from the corresponding author on reasonable request.
